# Advancing Undergraduate Student Mental Healthcare of Social Anxiety Disorder: Evaluating the Acceptance of AR-Assisted Cognitive Behavioral Therapy Through TAM-Based Constructs

**DOI:** 10.3390/healthcare14131978

**Published:** 2026-07-03

**Authors:** Zixuan Zhou, Yubo Zhou, Bo Ouyang, Siu Shing Man, Alan Hoi Shou Chan

**Affiliations:** 1 School of Design, South China University of Technology, Guangzhou 510006, China; 202330741752@mail.scut.edu.cn (Z.Z.); ssman6@scut.edu.cn (S.S.M.); 2 School of Computer Science, Beijing Institute of Technology, Beijing 100081, China; yubozhou547@gmail.com; 3 College of Engineering, City University of Hong Kong (Dongguan), Dongguan 523808, China; alan.chan@cityu.edu.hk

**Keywords:** AR technology, technology acceptance, undergraduate student, social anxiety disorder, personal factors

## Abstract

**Background**: As a crucial transitional period from campus to society, providing comprehensive undergraduate health psychological care is essential for addressing Social Anxiety Disorder (SAD). Current global healthcare research is actively exploring innovative digital interventions, with a specific focus on leveraging Augmented Reality (AR) as a transformative auxiliary tool in clinical settings. **Methods**: This study investigates the factors influencing the acceptance of AR-assisted Cognitive Behavioral Therapy (CBT) within student healthcare frameworks by developing a research model based on the Technology Acceptance Model (TAM). The framework incorporates key clinical and behavioral constructs: self-efficacy (SE), facilitating conditions (FC), and social influence (SI). **Results**: SE, FC, and SI significantly and positively impact the willingness to adopt AR technology for mental health purposes. Based on these findings, practical recommendations are provided for healthcare technology developers, therapists, and university psychological care providers to enhance the integration of AR-assisted CBT. **Conclusions**: Strengthening these digital pathways is vital for improving healthcare outcomes and enabling students to navigate future social and professional environments effectively. Because the sample consisted solely of Chinese undergraduate students, the findings should be interpreted within this specific cultural and educational context and require further validation in cross-cultural and multi-regional samples.

## 1. Introduction

University students with social anxiety disorder (SAD) experience significant and persistent fear of social situations within which they anticipate rejection, scrutiny, and embarrassment [1,2]. SAD is a common anxiety disorder characterized by marked fear or anxiety in social or performance situations in which individuals may be exposed to possible scrutiny by others [3]. Individuals with SAD often fear negative evaluation, embarrassment, rejection, or visible anxiety symptoms, which can lead to avoidance of social interaction, academic participation, public speaking, and other performance-related situations [4]. International epidemiological evidence indicates that SAD is not limited to a specific national or cultural context. Cross-national data showed that SAD is a globally distributed mental health condition, with substantial prevalence across countries and regions [5,6,7]. SAD also tends to emerge during adolescence and early adulthood, making university students a particularly relevant population for investigation [6]. As a common mental health condition, SAD significantly impairs social interactions, academic performance, and professional functioning in children, adolescents, and youth [8]. SAD could have a severe impact on individuals’ educational attainment and ability to work productively and maintain social relationships [9]. As a crucial transitional period from campus to society, ensuring robust undergraduate health psychological care is essential for addressing SAD [10]. In current intervention studies for SAD, cognitive behavioral therapy (CBT) was the most widely used treatment paradigm [11,12,13].

CBT is a time-limited and present-oriented approach to psychotherapy that equips patients with the mental and social tools necessary for adaptive functioning in their private lives and social circles [14]. Perera [15] argued that CBT is overly focused on immediate symptom reduction rather than addressing the deep and underlying personality structures or developmental traumas. Traditional CBT often struggles to address systemic stressors or diverse cultural values because its Western-centric framework primarily focuses on correcting an individual’s internal cognitive distortions [16]. Currently, because information technology continues to evolve, CBT is moving toward an intelligent direction [17]. How to effectively apply new technologies in CBT for SAD has become a widely discussed topic in the academic community [18]. Some studies combined internet technology with CBT and applied this approach to the treatment of SAD among undergraduate students [19]. Studies led by Menzies and colleagues showed that standalone and fully automated online CBT programs could deliver structured cognitive restructuring and exposure-based exercises without continuous face-to-face clinician contact [20]. For example, CBTpsych and iGlebe were developed to address social anxiety associated with stuttering and demonstrated reductions in social anxiety symptoms, unhelpful cognitions, and avoidance-related difficulties [21]. Similarly, web-based CBT programs such as Overcome Social Anxiety showed significant symptom improvement among users who completed the intervention [22]. These findings indicated that online CBT can improve access to evidence-based SAD treatment and support self-guided therapeutic engagement. Global therapeutic studies were actively exploring innovative strategies to aid in the treatment of SAD, with particular emphasis on leveraging augmented reality (AR) technology as an effective auxiliary therapeutic tool [23,24].

AR technology overlays simulated digital elements onto the physical world, allowing users to interact with 3D virtual objects while remaining grounded in their actual surroundings [25]. Generally categorized into marker-based, markerless, and location-based types, AR provides diverse mechanisms for integrating digital overlays [26]. Among these types, markerless AR offers superior flexibility and stability for dynamic environments [27]. While AR technology has been developed for various applications in healthcare and education, its use in a blended-reality approach is considered effective for administering CBT, particularly for SAD [28,29,30,31,32]. AR-assisted CBT enhances the treatment of SAD by providing a highly controllable and accessible medium for graded exposure therapy, allowing patients to confront anxiogenic stimuli in familiar environments [23]. The efficacy of this approach is predicated upon the seamless combination of real and virtual worlds, interactivity, and real-time registration [23]. Furthermore, studies suggested that AR-integrated CBT exerts a more favorable impact on patient treatment engagement and adherence than conventional in vivo exposure approaches [33]. AR technology significantly reduces people’s anxiety levels and increases tolerance for fear-inducing situations, providing a strong precedent for SAD research [34]. Employing AR technology for contextualized exposure positively influences patients by promoting their coping efficacy and enhancing their positivity for self-directed therapeutic practice [35]. Despite these promising clinical foundations, current research remains primarily focused on the clinical efficacy of AR-assisted CBT for undergraduate students with SAD, leaving an important research gap in understanding the behavioral factors that determine actual adoption.

From a resource-sustainability perspective, AR-assisted interventions offer a scalable alternative to traditional face-to-face therapy, reducing the demand for physical clinical space and minimizing the carbon footprint associated with travel to specialized centers. Beyond these ecological factors, the socio-economic impact of AR-assisted interventions encompasses multi-level cost savings and geographic liberation [36]. On a macroeconomic scale, deploying automated or cloud-based AR protocols alleviates the financial and human resource burdens on institutional wellness programs and government-funded public health sectors, fostering a more resilient healthcare ecosystem [37]. Individually, AR-assisted interventions eliminate geographic barriers, allowing undergraduate students in remote or underserved regions to access high-fidelity mental health support without being tethered to a specific centralized clinic [38]. Furthermore, the rapid transition from specialized hardware to cross-platform and markerless mobile AR vastly reduces platform dependency [39]. By enabling delivery through ubiquitous personal smartphones, AR-assisted interventions lower technological and financial barriers to entry, thereby ensuring high scalability and democratic access across diverse demographic strata.

There are many studies focusing on the TAM in the field of digital mental health. For instance, Nævdal et al. [40] examined therapists’ acceptance of internet-delivered cognitive behavioral therapy in Norway and constructed a TAM-based model to explain how therapist-related characteristics influenced the acceptance of mobile CBT in psychological treatment. Kelly et al. [41] investigated public acceptance of ChatGPT (Version 4o) for mental and physical healthcare and developed an extended TAM framework to explain users’ intention to adopt conversational AI as a healthcare-support technology. Jones et al. [42] evaluated the acceptance and usability of a virtual reality intervention among military members and veterans with treatment-resistant posttraumatic stress disorder, as well as therapists and VR operators. Although these studies have examined the TAM for different digital types of mental health treatments, limited research has specifically constructed a TAM-based model for AR-assisted psychotherapy among undergraduate students. Bouguettaya and Aboujaoude [43] found that group identification in social influence (SI) has a certain impact on students’ acceptance of AR-based therapy for anxiety disorders. Although Patel et al. [44] emphasized that SI is critical for understanding CBT-assisted AR technology adoption among adults with SAD, SI has not been rigorously analyzed through quantitative models in undergraduate populations. Moreover, Hazell et al. [45] found that facilitating conditions (FC), such as therapist control and environmental safety, are the primary reasons students chose technology-based interventions over real-world experiences, and comprehensively analyzed the reasons why students accept or reject digital therapeutics. Lau et al. [46] emphasized that FC and SE are the primary factors predicting whether students accept digital mental health interventions for depression and anxiety. While Chen et al. [47] highlighted a significant gap in understanding the link between students’ SE and digital therapeutic environments, inconvenient usage environments can undermine user confidence, reducing intentions to adopt the technology. Despite the growing integration of AR technology in clinical contexts, the complex interrelationships among SE, FC, SI, and behavioral intention remain insufficiently explored among undergraduate students using AR technology for therapeutic purposes, leaving an important research gap.

Accordingly, this study addressed the research gap by extending the technology acceptance model (TAM) with SE, FC, and SI to explain the acceptance of AR-assisted CBT among undergraduate students with SAD. By analyzing SE, FC, and SI, this study moved beyond clinical outcomes to identify the specific environmental and psychological factors required to transition AR-assisted CBT from an experimental auxiliary tool into a widely accepted and accessible standard of campus mental healthcare [48]. Ultimately, the findings of this study provided actionable insights for developers and therapists to optimize therapeutic environments and product design for increasing clinical adoption of AR-assisted interventions among undergraduate students with SAD.

## 2. Model Development and Hypotheses

### 2.1. Technology Acceptance Model

The TAM was originally developed by Davis [49] to predict the likelihood of a new technology being adopted by a group of people or an organization. The TAM included four important elements: perceived ease of use (PEOU), perceived usefulness (PU), attitude toward using (ATU), and behavioral intention to use (BI) [49]. Within the TAM framework, PU and PEOU are the core determinants of acceptance. Specifically, PEOU positively influences PU, and both factors significantly shape a user’s ATU. While ATU subsequently drives the BI to adopt the technology, PU is also theorized to have a direct impact on BI. The Technology Acceptance Model holds significant value for technology-assisted undergraduate health treatment, as it provides a structured psychological framework to evaluate how digital interventions transition from clinical tools to accepted patient practices [50,51]. PU referred to the level to which undergraduates believe that using AR-assisted CBT would help them alleviate SAD. PEOU referred to the degree to which undergraduates believe that using AR-assisted CBT would be effortless to operate. ATU referred to the undergraduate student’s overall positive or negative evaluative feeling toward AR-assisted CBT, representing the degree to which the student perceives the experience of using the technology as a favorable way to manage SAD. BI referred to undergraduate students’ subjective probability of using AR-assisted CBT. The TAM has been extensively used to explain students’ technology acceptance [52,53]. In addition, the TAM has been extensively used and extended in CBT to forecast people’s acceptance and use, such as in digital therapeutics [54] and internet-delivered interventions [40]. Drawing upon the TAM, we hypothesized that:

**H1.**  *PU positively affects ATU*.

**H2.**  *PEOU positively affects ATU*.

**H3.**  *PEOU positively affects PU*.

**H4.**  *ATU positively affects BI*.

**H5.**  *PU positively affects BI*.

### 2.2. Self-Efficacy

Self-efficacy (SE) refers to an individual’s belief in their capability to organize and execute the actions required to achieve specific goals [55]. In the present study, SE is defined as undergraduate students’ perceived confidence in their ability to use AR-assisted CBT for SAD, manage difficulties encountered during AR-based therapeutic activities, and persist in completing treatment-related tasks. Thus, SE does not represent students’ actual technical competence or the objective therapeutic effectiveness of AR-assisted CBT. Rather, SE reflects their subjective belief that they can successfully engage with the AR-assisted CBT process. In the context of digital mental health interventions, users with higher SE are more likely to approach technology-based treatment with confidence and persistence [56]. Moshe et al. [57] found that dropout was associated with users’ baseline characteristics and early engagement patterns, which also influence individuals’ SE because the characteristics and patterns can shape users’ confidence in their ability to engage with and successfully complete digital interventions. These findings highlighted the importance of SE and early user experiences in sustaining engagement from a technology acceptance perspective. SE was widely acknowledged as an essential predictor of students’ technology acceptance because it influences cognitive processing, emotional stability, and perceived ability to manage new technologies [58,59]. SE influenced undergraduate students’ cognitive processes and the intensity and stability of their emotional states when using new technology [58]. In addition, Larsen [60] conducted a review on TAM and found that SE was a factor that positively affects PU and PEOU. Drawing on this evidence, we proposed that:

**H6.**  *SE positively affects PEOU*.

**H7.**  *SE positively affects PU*.

### 2.3. Facilitating Conditions

FC encompass the objective elements in the environment that observers believe make an act easy to perform, specifically reflecting the perceived accessibility of the infrastructure and technical support systems required to utilize a new technology [61]. FC serves as a measure of students’ perceptions regarding the robustness of institutional resources, ranging from hardware availability to the immediacy of technical assistance [62]. For university students engaging with AR-based CBT, FC extend beyond basic connectivity to include specialized hardware and conducive on-campus physical environments. Empirical evidence underscored the importance of FC in technology acceptance. Also, FC significantly bolster PU and PEOU among clinicians delivering psychological interventions [63]. Because AR applications are resource-intensive, requiring high-bandwidth wireless networks and specialized technical frameworks often managed by campus IT and external providers, the presence of these supportive conditions is likely a foundational determinant in a university student’s decision to adopt such innovative therapeutic tools. Therefore, it was hypothesized that:

**H8.**  *FC positively affects PEOU*.

**H9.**  *FC positively affects PU*.

### 2.4. Social Influence

SI refers to the pressure an individual feels from important figures in their life to adopt the new system [61]. When individuals participate in a social system, they identify with and assume a role within that system and typically act in accordance with the expectations of other members [64]. According to Venkatesh and Davis [65], SI were broadly interpreted to include subjective norms (the pressure to conform to the expectations of important others) and image (the desire to maintain or enhance one’s social status within a group). For undergraduate students with SAD, SI include conforming to the expectations of those around them and establishing a positive social image. The way that SI involves social roles enhances undergraduate students’ PU within the context of using technology [66]. Specifically, interpersonal interaction generates perceived pressure through messages or signals for individuals to act on certain behaviors [67]. Extensive research indicated that SI exerted a significant positive effect on PEOU because endorsement from peers and mentors reduces the cognitive burden and anxiety associated with adopting new technology [68,69]. When undergraduate students perceive AR technology as socially validated and trend-relevant, they tend to view the AR technology as more approachable and less intimidating, effectively lowering the perceived barrier to entry [65]. Therefore, it was hypothesized that:

**H10.**  *SI positively affects PEOU*.

**H11.**  *SI positively affects PU*.

**Figure 1:**
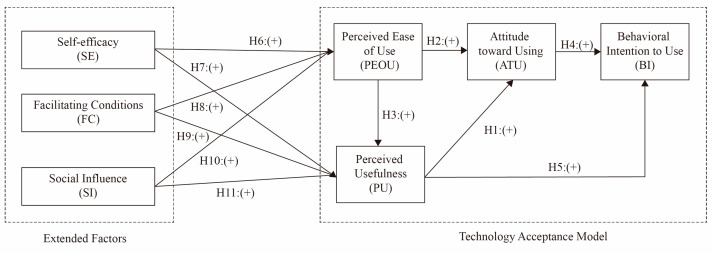
Research model for explaining the acceptance of AR-assisted CBT among undergraduate students with SAD.

Based on the relevant literature, this study developed a research model of the 11 hypotheses (Figure 1) to explain the acceptance of AR-assisted CBT among undergraduate students with SAD.

## 3. Methods

### 3.1. Participants

Participants were recruited using a purposive sampling strategy to ensure the study population consisted specifically of undergraduate students exhibiting clinical levels of social anxiety. The recruitment process was conducted at large public universities, where an initial invitation was disseminated via social media platforms. To minimize self-selection bias and ensure data integrity, a two-stage screening protocol was implemented. In the first stage, a total of 1000 students completed a preliminary demographic survey and the Social Phobia Inventory (SPIN) [70]. In the second stage, only those meeting the clinical threshold (SPIN score ≥ 19) were invited to participate in the full study regarding AR-assisted CBT acceptance. A total of 258 undergraduate students successfully completed the study. The sample consisted of 31.8% male and 68.2% female participants, with a mean age of 19.6 years (SD = 0.5). Regarding clinical status, all participants met the inclusion criteria for SAD with a mean SPIN score of 28.3 (SD = 3.1), indicating a moderate level of social anxiety across the cohort. In terms of technological proficiency, 76.7% of participants reported prior experience with AR devices, primarily via mobile-based applications. The duration of previous AR use was relatively distributed, with the majority (79.8%) of experienced users having utilized AR technology for less than three months. All participants provided informed consent, and the study was conducted in accordance with the Declaration of Helsinki.

### 3.2. Measurements

Prior to the assessment of technology acceptance, participants were introduced to an AR-assisted CBT protocol designed to alleviate social anxiety symptoms through immersive digital exposure [71]. The protocol was standardized and smartphone-based with cross-platform AR functions and markerless environmental tracking to detect spatial surfaces and anchor virtual classroom environments, digital avatars, and holographic prompts in real time. Participants accessed the protocol on their personal Android or iOS smartphones and interacted with simulated social scenarios and cognitive restructuring prompts through touch gestures, providing a safe, controlled, scalable, and repeatable medium for exposure therapy [72,73]. These contents were selected because AR allows for the systematic manipulation of social threat levels, such as varying the audience’s gaze or facial expressions, which enhances ecological validity while maintaining the safety of a clinical setting. Each session was designed to last 20 to 30 min, with a recommended total duration of four to six weeks (two sessions per week), allowing for gradual habituation to social stressors and the reinforcement of SE through successful virtual interactions.

To evaluate the research hypotheses, empirical data were collected via a self-administered questionnaire structured into three comprehensive sections. The initial portion of the instrument captured essential demographic characteristics, such as age and gender, while simultaneously establishing a clinical baseline for the study. To ensure a standardized and professional identification of SAD among the undergraduate cohort, participants were screened using the Social Phobia Inventory (SPIN). This 17-item self-report scale assesses dimensions of fear, avoidance, and physiological symptoms. Following the established methodology of Connor et al. [74], a cut-off score of 19 or higher was implemented to distinguish individuals with clinically significant symptoms from the broader student population. The second phase of the survey focused on participants’ acceptance of AR-assisted CBT by operationalizing the core components of TAM, specifically PU, PEOU, ATU, and BI. These measurement items were refined following an extensive review of existing literature to ensure they were contextually relevant to the unique intersection of AR and therapeutic intervention for SAD. Building upon this, the final section examined the specific antecedents of technology adoption: SE, FC, and SI. These constructs were measured using 21 items adapted from the work of Zhang et al. [75] and Chung and Dong [76], with each factor represented by three specific indicators. As detailed in Table 1, which lists the content of items with their corresponding references, all responses were gathered using a five-point Likert scale ranging from “strongly disagree (1)” to “strongly agree (5).” Pilot testing confirmed the practical feasibility of the instrument, with the average completion time recorded at approximately 10 min.

### 3.3. Data Analysis

According to Anderson and Gerbing [82], the structural equation modeling (SEM) has two steps. The first step involved carrying out confirmatory factor analysis (CFA) to test the measurement’s psychometric properties and verify the reliability and validity of the research instrument. In the subsequent step, the developed hypotheses were tested with SEM. AMOS 26 was used to conduct CFA and SEM, with model fit evaluated in accordance with goodness-of-fit criteria [83]. These goodness-of-fit metrics included the chi-square to degrees of freedom ratio (*χ*^2^/*df*), comparative fit index (CFI), standardized root mean square residual (SRMR), and root mean square error of approximation (RMSEA). As suggested by Hu and Bentler [84], a model exhibits a good fit when *χ*^2^/*df* < 5, CFI > 0.90, SRMR < 0.08, and RMSEA < 0.08. To examine consistency across multiple indicators corresponding to the same construct, the convergent validity of the measurement was evaluated by computing the average variance extracted (AVE) index [85]. An AVE value exceeding 0.5 is regarded as indicative of sufficient convergent validity. To determine the discriminant validity of the measurement, the square root of AVE (SAVE) was calculated for each construct [86]. Specifically, the SAVE value for each factor ought to be higher than any bivariate correlation involving that factor in the model. Following the guidelines proposed by Cronbach [87] for measuring internal consistency reliability, Cronbach’s alpha coefficient was computed for each construct. A Cronbach’s alpha value greater than 0.7 indicates that the measurement demonstrates acceptable internal consistency reliability.

## 4. Results

### 4.1. Measurement Model Assessment

CFA results (Table 2) showed that the factor loading (FL) and composite reliability (CR) of items were larger than the critical value of 0.7, and the AVE for each construct was larger than the critical value of 0.5, which indicated that the measurement model exhibits adequate and robust convergent validity. The Cronbach’s alpha values for all constructs were greater than the critical requirement of 0.7 (ranging from 0.912 to 0.979), implying a high level of internal consistency reliability across the latent constructs. In Table 3, all constructs presented SAVE values that surpassed their respective inter-construct bivariate correlations, showing that the discriminant validity of the measurement was supported. Collectively, the measurement model yielded acceptable levels of model fit, validity, and reliability for structural equation modeling analysis. In addition, as shown in Table 4, the measurement model showed an acceptable fit to the data. Specifically, *χ*^2^/*df* = 3.762 was below the recommended threshold of 5, CFI = 0.952 exceeded the recommended value of 0.90, and both SRMR = 0.064 and RMSEA = 0.058 were below the recommended cut-off value of 0.08. These results indicated that the measurement model satisfied the commonly accepted model fit criteria.

### 4.2. Structural Model Assessment

The structural models were evaluated using identical model fit indices as those employed in the evaluation of the measurement models. The specific data of the model fitting indicators are as follows. The *χ*^2^/*df* was 3.871, which is below the recommended threshold of 5 [88]. The CFI was 0.945, exceeding the minimum acceptable value of 0.90. The SRMR was 0.068, below the 0.08 benchmark. The RMSEA was 0.064, falling within the acceptable range of less than 0.08. The obtained model fit indices reached the recommended thresholds, providing empirical evidence that the structural model sufficiently reflects the hypothesized relationships. Table 5 indicates the results of the hypothesis testing. All 11 hypotheses were supported empirically. SE, FC, and SI positively influenced PU and PEOU. PEOU positively influenced PU and ATU. PU positively influenced ATU and BI. ATU positively influenced BI.

## 5. Discussions

To address the limitations in existing research, this study constructed an extended TAM that incorporates SE, FC, and SI to explain undergraduate students’ acceptance of AR-assisted CBT for SAD. The findings revealed that undergraduate students’ acceptance of AR-assisted CBT for SAD was influenced by SE, FC, and SI. The data of 258 undergraduate students with SAD demonstrated the effectiveness of this model in clarifying the acceptance of AR-assisted CBT techniques among this group. This study provides theoretical, practical contributions, limitations, and future research opportunities for enhancing the adoption of AR-assisted CBT techniques among undergraduate students with SAD.

### 5.1. Theoretical Implications

This study extended the TAM by integrating SE, FC, and SI to explain undergraduate students’ acceptance of AR-assisted CBT for SAD. The results supported the core TAM pathways, showing that PEOU positively influenced PU and ATU, PU positively influenced ATU and BI, and ATU positively influenced BI. These findings were consistent with the original TAM [49] and previous AR technology acceptance studies [89,90]. They also aligned with evidence from mobile learning research showing that positive attitudes can strengthen students’ intention to use new technologies [91]. Therefore, the present study provided preliminary theoretical support for applying the TAM to the acceptance of AR-enabled mental health interventions among undergraduate students with SAD [92].

The findings also showed that SE positively influenced both PEOU and PU. This finding suggested that undergraduate students who felt more confident in their ability to manage AR-assisted CBT tasks were more likely to perceive the system as easy to use and useful. This result was consistent with previous studies indicating that SE is an important factor in healthcare technology acceptance [66,93] and students’ technology acceptance [58,94,95]. SE may reduce uncertainty when students interact with AR interfaces, allowing them to focus more on therapeutic activities, such as simulated social exposure and cognitive restructuring, rather than on the complexity of the technology itself [96,97,98,99].

In addition, FC and SI were significant antecedents of PEOU and PU. The positive effects of FC are consistent with previous findings showing that technical and environmental support can improve users’ perceptions of usefulness and ease of use in psychological intervention and AR-related contexts [62,100]. In the context of AR-assisted CBT, accessible devices, stable network conditions, appropriate usage environments, and timely technical support may reduce perceived barriers and improve students’ confidence in using the system [101,102]. Similarly, the positive effects of SI are consistent with prior research indicating that social expectations, peer endorsement, and collective support can shape users’ acceptance of new technologies [61,103,104,105,106]. Overall, these findings suggested that acceptance of AR-assisted CBT is shaped by a combination of personal confidence, environmental support, and social influence. Given that the sample consisted of Chinese undergraduate students with SAD, the conclusions should be interpreted within this cultural and demographic context, and their generalizability to other populations requires further validation.

### 5.2. Practical Implications

According to the findings of this study, practical recommendations were proposed to enhance undergraduate students’ acceptance of AR-assisted CBT for SAD, thereby helping more undergraduate students alleviate social anxiety to cope with future social life. First, developers and clinicians should strengthen students’ SE by providing a short, low-stakes onboarding process before formal AR-assisted CBT use. For example, students could first practice simple AR interactions in neutral scenarios before entering socially evaluative exposure tasks [107,108,109]. Clear instructions, real-time progress feedback, and error-recovery functions may help reduce uncertainty and reinforce students’ belief that they can manage the AR system effectively [110]. These design features may improve both PEOU and PU by making the technology feel more manageable and therapeutically relevant. Second, universities and counseling centers should improve FC by ensuring that students can access the specific resources needed to use smartphone-based AR-assisted CBT. Therefore, when developing the technology, it should be ensured that the AR treatment platform can be used compatibly with the existing infrastructure in the campus, and that users can contact trained staff for assistance when problems arise [111,112]. These conditions are directly related to the usability and accessibility of AR-assisted CBT and may reduce technical barriers that could otherwise weaken students’ PEOU and PU. Third, SI had a positive impact on PU and PEOU. This finding suggests that students’ acceptance of AR-assisted CBT may be shaped by the attitudes, recommendations, and perceived approval of important others, including peers, therapists, university counselors, and family members. For example, recommendations and encouragement from companions, family members, and friends may increase students’ familiarity with AR-assisted CBT, enhance its perceived credibility, and strengthen perceived usefulness and perceived ease of use in SAD management [33,69]. Finally, developers should enhance PU and PEOU by designing AR-assisted CBT systems that are simple to operate, relevant to students’ social contexts, and sensitive to privacy and stigma. Incorporating realistic campus scenarios, visual biofeedback, intuitive interaction methods, and natural gesture-based controls may improve students’ perceptions of usefulness and ease of use [113,114,115]. In addition, reducing overly clinical visual cues and adopting a health-oriented interface design may help lower stigma and support more positive attitudes toward AR-assisted CBT [116]. These recommendations should be further validated in future empirical studies before being translated into routine therapeutic practice. The proposed suggestions provide initial guidance for improving the acceptance of AR-assisted CBT among undergraduate students with SAD. However, they should be further tested through controlled experimental studies, longitudinal field evaluations, and clinical trials before being translated into large-scale therapeutic practice.

### 5.3. Limitations and Future Research Opportunities

This study must acknowledge several research limitations. First, the sample of this study was limited to participants recruited in China, which may lead to the restricted generalizability of the findings. Regional differences in cultural, social, and economic contexts have been shown to substantially influence technology acceptance behaviors. Therefore, to address the potential generalizability constraints inherent in the present study’s regional sample, subsequent research should validate the findings using multi-regional data to establish the model’s applicability across diverse populations. Comparative cross-national investigations are particularly well-suited to illuminate the role of cultural factors in shaping AR-assisted CBT acceptance, thereby enabling the development of culturally tailored frameworks for global AR-assisted CBT promotion. Second, acceptance was often measured at a single point in time (cross-sectional). For AR-assisted CBT, acceptance might fluctuate as the undergraduate students become more habituated to the hardware or as their anxiety levels change throughout the semester [117]. Therefore, future research could consider incorporating longitudinal study methods to investigate the dynamic changes and influencing factors of undergraduate students regarding AR-assisted CBT for SAD in depth. Third, the research model of this study primarily focused on positive antecedents derived from classic technology acceptance paradigms. While this directional formulation aligned with established literature, it inherently omitted potential counter-forces that could inhibit technology adoption [118]. Future research should strive for a balanced and bidirectional theoretical framework by integrating negative factors, such as technology anxiety, privacy concerns, or psychological resistance. Examining how these negative nodes interact with self-efficacy and facilitating conditions can provide a nuanced and comprehensive diagnostic tool for institutional stakeholders deploying digital therapeutics. Fourth, the questionnaire items were not newly developed in the present study but were adapted from established technology acceptance and digital health acceptance studies [49,119]. All Likert-scale items in the questionnaire were positively worded. Although the items were adapted from established technology acceptance instruments and demonstrated acceptable reliability and validity in the present study, the exclusive use of positively framed statements may have increased the possibility of acquiescence bias or agreement tendency. Therefore, future research should further refine the questionnaire design by considering balanced item wording, attention-check items, common method bias testing, or comparison between positively worded and mixed-wording scales [120]. Fifth, the sample consisted of undergraduate students recruited from large public universities, a highly tech-savvy cohort. In addition, 76.7% of participants reported prior AR experience, primarily through mobile-based applications. Therefore, the study population may represent a relatively technology-experienced cohort, which may have increased their PEOU, PU, ATU, and BI. This characteristic may limit the generalizability of the findings to less digitally experienced students, older adults, patients from non-university settings, or populations with limited access to digital technologies. Future research should recruit participants with more diverse levels of digital literacy, AR familiarity, and technology access to further validate the proposed model across broader healthcare populations. Sixth, the sample of this study consisted of 31.8% male and 68.2% female participants, indicating that female undergraduate students were overrepresented. Prior technology-acceptance research suggested that gender may shape how users evaluate new technologies, including the relative importance of perceived usefulness, perceived ease of use, and social influence [121]. The gender imbalance of the sample in this study may have influenced the estimated acceptance of AR-assisted CBT. Future research should recruit a gender-balanced sample and examine whether the structural relationships in the proposed model differ between male and female students.

## 6. Conclusions

This study integrated TAM, SE, FC, and SI to propose a research model explaining the acceptance of AR-assisted CBT for SAD among undergraduate students. The results indicated that TAM effectively explains undergraduate students’ acceptance of AR-assisted CBT for SAD. SE, FC, and SI were all confirmed as positive influencing factors of PU and PEOU. This study emphasized the importance of understanding undergraduate students’ acceptance of AR-assisted CBT for SAD and enriched the relevant literature. Findings generated by the present research enriched the current body of AR-assisted CBT and offered fundamental theoretical support for SAD therapists, AR-based SAD treatment developers, and stakeholders. These findings provide preliminary theoretical evidence for understanding acceptance-related factors in AR-assisted CBT and may offer useful reference value for SAD therapists, university mental health providers, and digital health developers. Furthermore, the results offer actionable insights for the design of AR-assisted healthcare interventions, assisting developers in implementing measures that streamline undergraduate health psychological care. The research results could assist developers of AR-assisted CBT in designing AR models and implementing measures to help undergraduate students treat SAD, thereby facilitating their adaptation to social life.

## Figures and Tables

**Table 1:** Item contents and respective references.

Construct	Item	Content	Reference
Perceived Ease of Use (PEOU)	PEOU1	1. Using AR to assist CBT in the treatment of SAD was easy for me.	[77]
	PEOU2	2. Using the AR system was easy for me.	
	PEOU3	3. Learning how to use the AR system was easy for me.	
Perceived Usefulness (PU)	PU1	1. Using AR to assist CBT would be useful for the treatment of SAD.	[78]
	PU2	2. Using AR to assist CBT would make the treatment of SAD more effective.	
	PU3	3. Using AR to assist CBT would improve my social performance.	
Self-efficacy (SE)	SE1	1. I am confident I can solve problems when I use AR to assist CBT in the treatment of SAD.	[79]
	SE2	2. When I encounter difficulties, I can usually devise some solutions to address them.	
	SE3	3. I can face difficulties calmly because I trust in my ability.	
Facilitating Conditions (FC)	FC1	1. I have the resources necessary to use AR to assist CBT in the treatment of SAD.	[80]
	FC2	2. I have the knowledge necessary to use AR to assist CBT in the treatment of SAD.	
	FC3	3. I can get help from others when I have difficulties using AR to assist CBT in the treatment of SAD.	
Social Influence (SI)	SI1	1. People who are important to me think I should use AR to assist CBT in the treatment of SAD.	[80]
	SI2	2. People who influence my behavior believe that I should use AR to assist CBT in the treatment of SAD.	
	SI3	3. People whose opinions I value prefer me to use AR to assist CBT in the treatment of SAD.	
Attitude toward Using (ATU)	ATU1	1. Using AR to assist CBT in the treatment of SAD is a good idea.	[78]
	ATU2	2. Using AR to assist CBT in the treatment of SAD is a wise idea.	
	ATU3	3. I feel positive about using AR to assist CBT in the treatment of SAD.	
Behavioral Intention to Use (BI)	BI1	1. Assuming I can use AR to assist CBT in the treatment of SAD, I intend to use it.	[81]
	BI2	2. Given that I can use AR to assist CBT in the treatment of SAD, I predict that I will use it.	
	BI3	3. If I can use AR to assist CBT in the treatment of SAD, I would like to use it as much as possible.	

Note: PEOU: Perceived Ease of Use; PU: Perceived Usefulness; SE: Self-efficacy; FC: Facilitating Conditions; SI: Social Influence; ATU: Attitude toward Using; BI: Behavioral Intention to Use.

**Table 2:** CFA results, convergent validity, and internal consistency.

Construct	Item	Mean	SD	FL	AVE	CR	Cronbach’s Alpha
Perceived Ease of Use (PEOU)	PEOU1	3.923	0.913	0.863	0.799	0.952	0.950
	PEOU2	3.955	0.926	0.932			
	PEOU3	3.894	0.831	0.886			
Perceived Usefulness (PU)	PU1	3.786	0.913	0.892	0.738	0.915	0.913
	PU2	3.724	0.926	0.815			
	PU3	3.933	0.974	0.868			
Self-efficacy (SE)	SE1	3.874	0.852	0.931	0.828	0.966	0.965
	SE2	3.951	0.914	0.884			
	SE3	3.487	0.923	0.915			
Facilitating Conditions (FC)	FC1	3.564	1.011	0.917	0.870	0.981	0.979
	FC2	3.595	0.982	0.924			
	FC3	3.783	0.953	0.957			
Social Influence (SI)	SI1	3.924	0.869	0.871	0.756	0.927	0.925
	SI2	3.868	0.914	0.889			
	SI3	3.916	0.847	0.848			
Attitude toward Using (ATU)	ATU1	3.835	0.831	0.914	0.817	0.961	0.960
	ATU2	3.794	0.883	0.901			
	ATU3	3.843	0.948	0.897			
Behavioral Intention to Use (BI)	BI1	3.769	0.932	0.859	0.737	0.914	0.912
	BI2	3.944	0.966	0.834			
	BI3	3.853	1.029	0.881			

Note: PEOU: Perceived Ease of Use; PU: Perceived Usefulness; SE: Self-efficacy; FC: Facilitating Conditions; SI: Social Influence; ATU: Attitude toward Using; BI: Behavioral Intention to Use; SD: Standard Deviation; FL: Factor Loading; AVE: Average Variance Extracted; CR: Composite Reliability; Cronbach’s alpha: Internal consistency reliability coefficient.

**Table 3:** Correlations among constructs.

	SE	FC	SI	PEOU	PU	ATU	BI
SE	0.894						
FC	0.564	0.859					
SI	0.435	0.659	0.910				
PEOU	0.759	0.684	0.524	0.933			
PU	0.592	0.725	0.692	0.814	0.869		
ATU	0.685	0.633	0.742	0.651	0.782	0.904	
BI	0.741	0.670	0.769	0.646	0.842	0.825	0.858

Note: Values on the diagonal are the SAVE of the constructs. Values below the diagonal are the correlations among the constructs. SE: Self-efficacy; FC: Facilitating Conditions; SI: Social Influence; PEOU: Perceived Ease of Use; PU: Perceived Usefulness; ATU: Attitude toward Using; BI: Behavioral Intention to Use; SAVE: Square Root of Average Variance Extracted.

**Table 4:** Results of model fit indices for the measurement model.

Model Fit Indices	Model	Recommended Values	Results	References
*χ*^2^/*df*	3.762	<5	Acceptable	[88]
CFI	0.952	≥0.9	Acceptable	
SRMR	0.064	<0.08	Acceptable	
RMSEA	0.058	<0.08	Acceptable	

Note: *χ*^2^/*df*: Chi-square to degrees of freedom ratio; CFI: Comparative Fit Index; SRMR: Standardized Root Mean Square Residual; RMSEA: Root Mean Square Error of Approximation.

**Table 5:** Results of hypothesis testing.

Hypothesis	Standardized Path Coefficient	*p*-Value
H1: PU → ATU.	0.426	<0.001
H2: PEOU → ATU.	0.382	<0.001
H3: PEOU → PU.	0.514	<0.001
H4: ATU → BI.	0.538	<0.001
H5: PU → BI.	0.561	<0.001
H6: SE → PEOU.	0.412	<0.001
H7: SE → PU.	0.325	<0.001
H8: FC→ PEOU.	0.348	<0.001
H9: FC → PU.	0.370	<0.001
H10: SI → PEOU.	0.287	<0.001
H11: SI → PU.	0.354	<0.001

Note: PU: Perceived Usefulness; ATU: Attitude toward Using; PEOU: Perceived Ease of Use; BI: Behavioral Intention to Use; SE: Self-efficacy; FC: Facilitating Conditions; SI: Social Influence.

## Data Availability

The data presented in this study are available on request from the corresponding author due to personal privacy protection and ethical research requirements.

## Abbreviations

The following abbreviations are used in this manuscript:

AR	Augmented reality
ATU	Attitude toward using
AVE	Average variance extracted
BI	Behavioral intention to use
CBT	Cognitive behavioral therapy
CFA	Confirmatory factor analysis
CFI	Comparative fit index
CR	Composite reliability
FC	Facilitating conditions
FL	Factor loading
PEOU	Perceived ease of use
PU	Perceived usefulness
RMSEA	Root mean square error of approximation
SAD	Social anxiety disorder
SAVE	Square root of average variance extracted
SE	Self-efficacy
SEM	Structural equation modeling
SI	Social influence
SPIN	Social Phobia Inventory
SRMR	Standardized root mean square residual
TAM	Technology acceptance model
